# Supplementary material for: Complexome profiling of the Chlamydomonas psb28 mutant reveals TEF5 as an early PSII assembly factor
Source: Plant Cell. 2025 Mar 17;37(6):koaf055. doi: 10.1093/plcell/koaf055 (PMC12164590; doi:10.1093/plcell/koaf055)
Supplement: koaf055_Supplementary_Data [file koaf055_supplementary_data.zip › psb28_interactive.zip!/plots/intensity/qualityPlots/peptides/_Cre12.g503350.t1.2.html]

### Cre12.g503350.t1.2

Trivial name:   
SeqCoverage [median]: 19.70  
Mass: 25.38 kDa  
  
 

| Sequence | MissedCleavage | Mass [Da] | wtIntensity | muIntensity | Score |
| --- | --- | --- | --- | --- | --- |
| FQALEYQTSELKK | 1 | 1583.82 | 0.00 | 975.67 | 98.11 |
| LGFPQSVLPGEDER | 0 | 1542.77 | 0.00 | 448.26 | 54.47 |
| SAPAQLLLQASR | 0 | 1253.71 | 723.17 | 2005.67 | 76.52 |
| TGNTFLFK | 0 | 926.49 | 0.00 | 1541.48 | 76.48 |
|  |  |  | 723.2 | 4971.1 |  |
